# Assessing Time-Resolved fNIRS for Brain-Computer Interface Applications of Mental Communication

**DOI:** 10.3389/fnins.2020.00105

**Published:** 2020-02-18

**Authors:** Androu Abdalmalak, Daniel Milej, Lawrence C. M. Yip, Ali R. Khan, Mamadou Diop, Adrian M. Owen, Keith St. Lawrence

**Affiliations:** ^1^Department of Medical Biophysics, Western University, London, ON, Canada; ^2^Imaging Program, Lawson Health Research Institute, London, ON, Canada; ^3^Imaging Research Laboratories, Robarts Research Institute, London, ON, Canada; ^4^Brain and Mind Institute, Western University, London, ON, Canada

**Keywords:** functional near-infrared spectroscopy, brain-computer interface, motor-imagery, disorders of consciousness, time-resolved measurement

## Abstract

Brain-computer interfaces (BCIs) are becoming increasingly popular as a tool to improve the quality of life of patients with disabilities. Recently, time-resolved functional near-infrared spectroscopy (TR-fNIRS) based BCIs are gaining traction because of their enhanced depth sensitivity leading to lower signal contamination from the extracerebral layers. This study presents the first account of TR-fNIRS based BCI for “mental communication” on healthy participants. Twenty-one (21) participants were recruited and were repeatedly asked a series of questions where they were instructed to imagine playing tennis for “yes” and to stay relaxed for “no.” The change in the mean time-of-flight of photons was used to calculate the change in concentrations of oxy- and deoxyhemoglobin since it provides a good compromise between depth sensitivity and signal-to-noise ratio. Features were extracted from the average oxyhemoglobin signals to classify them as “yes” or “no” responses. Linear-discriminant analysis (LDA) and support vector machine (SVM) classifiers were used to classify the responses using the leave-one-out cross-validation method. The overall accuracies achieved for all participants were 75% and 76%, using LDA and SVM, respectively. The results also reveal that there is no significant difference in accuracy between questions. In addition, physiological parameters [heart rate (HR) and mean arterial pressure (MAP)] were recorded on seven of the 21 participants during motor imagery (MI) and rest to investigate changes in these parameters between conditions. No significant difference in these parameters was found between conditions. These findings suggest that TR-fNIRS could be suitable as a BCI for patients with brain injuries.

## Introduction

Brain-computer interfaces (BCIs) are devices that can be used to establish a communication pathway between the brain and external devices (Shih et al., 2012). For people with chronic paralysis following a severe spinal cord injury, surgical implants that record activity directly from the brain can provide a means of interacting with the environment, such as controlling a prosthetic (Mak and Wolpaw, 2009; Shih et al., 2012). However, the need to implant electrodes limits the applications of this invasive approach (Waldert, 2016). The use of neuroimaging modalities as non-invasive BCI devices has garnered attention for applications such as assessing cognition in patients with disorders of consciousness (DOC), providing rudimentary communication for patients in a completely locked-in state, and as a feedback tool for stroke therapy (Naseer and Hong, 2015a; Kurz et al., 2018; Rupawala et al., 2018). The most frequently used portable BCI devices are based on electroencephalography (EEG). Although EEG provides excellent temporal resolution, making it ideal for real-time applications, the technology suffers from poor spatial resolution and an inherent sensitivity to motion artifacts (Padfield et al., 2019). Motion artifacts can have an impact on the spectral content of EEG in the frequency range below 20 Hz and lead to large spikes in the signal that may be difficult to correct (Mihajlovic et al., 2014). A promising alternative is functional near-infrared spectroscopy (fNIRS) (Rupawala et al., 2018) since it provides a good compromise between spatial and temporal resolution.

Analogous to functional magnetic resonance imaging (fMRI), fNIRS detects increases in neuronal activity through the hemodynamic response — that is, the change in blood oxygenation that occurs due to increased cerebral blood flow (Monti et al., 2010). By measuring light absorption at a minimum of two wavelengths, changes in concentrations of oxy- and deoxy-hemoglobin can be calculated (Strangman et al., 2002). A number of activation paradigms have been combined with fNIRS for BCI applications, including motor imagery (MI), mental arithmetic, working memory, and other mental activities (Naseer and Hong, 2015a; Rupawala et al., 2018). MI was the first task proposed for BCI applications, which requires participants to perform kinesthetic imagining, such as imagining squeezing a ball (Coyle et al., 2004), finger tapping (Sitaram et al., 2007), and hand grasping (Fazli et al., 2012). More recent fNIRS-BCI applications have focused on activation paradigms that involve the prefrontal cortex, such as mental arithmetic, to avoid signal loss due to the presence of hair and concerns regarding the quality of the NIRS signal for MI tasks (Shin et al., 2017; Qureshi et al., 2017). However, MI has proven extremely valuable in fMRI studies of DOC. Using tennis imagery as a mental task and focusing on activation in the supplementary motor area (SMA), fMRI was used to demonstrate residual brain function in a patient with a diagnosis of vegetative state (Owen et al., 2006) and in a subsequent study, to provide “yes” and “no” answers to a series of questions (Monti et al., 2010).

To improve the sensitivity of fNIRS to MI, time-resolved (TR) fNIRS has been investigated (Abdalmalak et al., 2017a, 2020). TR detection involves recording the arrival times of single photons, which can be used to enhance depth sensitivity since photons that interrogate superficial tissue are detected earlier than photons that travel farther (i.e., deeper). Consequently, improved sensitivity to the brain can be achieved by focusing on late-arriving photons (Diop and St Lawrence, 2013; Lange and Tachtsidis, 2019). This can be achieved by calculating the statistical moments of the recorded distribution of arrival times since higher moments are weighted toward late-arriving light (Liebert et al., 2004; Milej et al., 2015). Previous work has shown that the first moment (i.e., the mean time-of-flight, <*t*>) provided a good compromise between depth sensitivity and signal-to-noise for detecting MI activation from probes interrogating the SMA and premotor cortex (PMC) (Abdalmalak et al., 2017a). Using fMRI as a benchmark, the classification accuracy of TR NIRS based on <*t*> analysis was 93% (Abdalmalak et al., 2017a). In a follow-up study, rudimentary communication was established with a locked-in patient who was instructed to use tennis imagery as affirmation to a series of questions (Abdalmalak et al., 2017b). The accuracy of the fNIRS-BCI responses was confirmed because the patient had regained sufficient eye movement to answer the same questions after the fNIRS study.

The promising results of the two previous studies suggest that time-resolved functional near-infrared spectroscopy (TR-fNIRS) combined with MI could be a suitable BCI for mental communication with DOC patients. The purpose of this study was therefore to evaluate the classification performance of this BCI approach on healthy volunteers. Each participant was asked a series of four questions requiring yes-or-no answers. They were instructed to imagine playing tennis to communicate “yes” and to stay relaxed if the answer was “no”(Monti et al., 2010). Linear discriminant analysis (LDA) and support vector machine (SVM) algorithms were evaluated for classification accuracy as these are the most commonly used machine-learning approaches used in fNIRS-BCI studies (Naseer and Hong, 2015a).

## Materials and Methods

### BCI Study

Twenty-one healthy participants with no history of any neurological disease were recruited (6 females and 15 males, mean age of 29 ± 5 years, age range 24–40 years). All participants except one were right handed with no history of neurological condition or severe brain injury. Written informed consent was obtained from all participants and this study was approved by the Research Ethics Board at Western University, which complies with the guidelines of the Tri-Council Policy Statement (TCPS): Ethical Conduct for Research Involving Humans.

**FIGURE 1:**
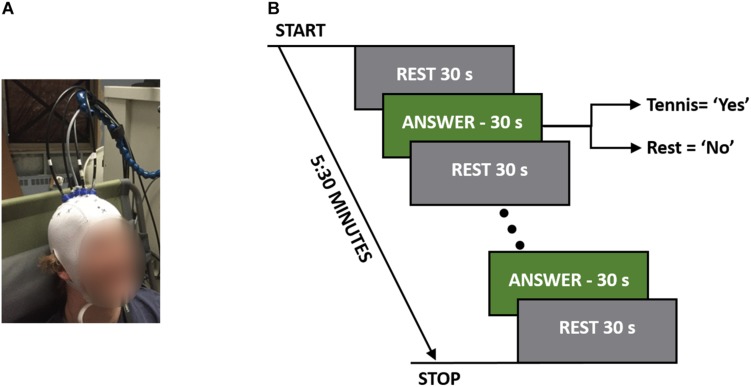
**(A)** A participant wearing the TR-fNIRS cap with the probes positioned over the SMA and PMC. **(B)** Study protocol illustrating the rest and response periods. The total time per question was 5:30 min, which consisted of five 30-s answer periods.

For each experiment, the participants were seated in a Fowler’s position on a reclining chair with a cushioned pillow to support their neck. The TR system consisted of one emission and four detection fibers (see section “TR-NIRS System”), which were placed on the head in a cross pattern with the emission fiber over FCz (according to the international template for EEG electrode placement) in order to interrogate the SMA and PMC (Abdalmalak et al., 2016). The fibers were secured on the head using a 3D printed holder (TAZ 5, LulzBot, United States), which was covered by an EEG cap (EASYCAP GmbH, Germany). Figure 1B shows a picture of one of the participants wearing the cap with the TR-NIRS optodes inserted.

Each participant was asked the following four questions that could all be answered with a “yes” or “no” response:

1. Do you have any brothers?
2. Do you have any sisters?
3. Are you at St. Joseph’s Hospital?
4. Are you feeling cold right now?

The order that questions were asked in was randomized between participants to avoid any biases that may exist based on the questions. These questions were chosen for their applicability to patient studies. For instance, the first two questions were factual with definitive known answers, while question 3 (“Are you at St. Joseph’s Hospital?) served as a control since all participants were expected to answer “yes.” The final question was chosen to simulate asking patients a question where only they would know the answer. Each question was asked five times in a block design consisting of a 30-s baseline rest period followed by five cycles of 30-s alternating blocks of “answer” and “rest” periods for a total duration of 5:30 min (Figure 1A). Answering all four questions took 22 min to complete. Each question was asked prior to the beginning of the run, and during the experiment the participants were cued to either “rest” or “answer.” For a positive response, the participants were asked to imagine playing a game of tennis where they pictured themselves on a tennis court, swinging their arm back and forth trying to hit a tennis ball over and over again. For a negative response, the participants were asked to remain completely relaxed; i.e., a “no” response would result in 5:30 min of complete rest.

### Physiological Monitoring Study

Since previous work has shown that changes in physiological variables such as heart rate (HR) and mean arterial pressure (MAP) can confound the fNIRS signal (Tachtsidis and Scholkmann, 2016), a subset (seven of the 21) of the participants were brought back for a separate session to investigate if the MI paradigm would elicit changes in HR and MAP. A non-invasive monitoring system was secured to the participant’s left arm (Finapres Medical Systems, Netherlands) to record HR and MAP continuously (sampling rate = 200 Hz) during a 5:30 min experiment consisting of 30-s alternating blocks of rest and MI. The cues given to the participants were similar to those given in the BCI study, except in this experiment, the participants were asked to imagine playing tennis every time they heard the word “tennis.”

For each participant, the Finapres data were subsequently down-sampled to 1 Hz and analyzed by averaging the data across each of the five MI and rest blocks. This resulted in five HR and five MAP values for each condition per participant. A paired *t*-test was used to determine if there was a significant difference between the two conditions across all participants while correcting for multiple comparisons using Bonferroni.

### TR-NIRS System

Data were collected using an in-house built TR-fNIRS system (Milej et al., 2016b; Kewin et al., 2019). The system consisted of two lasers (λ = 760 and 830 nm) pulsing at 80 MHz and controlled by a Sepia laser driver (PicoQuant, Germany). The laser heads were coupled in a 2.5 m bifurcated fiber (φ = 0.4 mm, NA = 0.39, Thorlabs, United States) and four 1.5 m detection fiber bundles (φ = 3.6 mm, NA = 0.55, Fiberoptics Technology, United States) were used to deliver the diffusively reflected light from the scalp to one of four hybrid photomultiplier tubes (PMA Hybrid 50, PicoQuant, Germany). A time-correlated single-photon counting module (HydraHarp 400, PicoQuant, Germany) was used to record the distribution of times-of-flight (DTOF) of photons for each detector every 300 ms using in-house-developed LabVIEW (National Instruments, United States) software (Milej et al., 2016a).

### TR-fNIRS Data Analysis

Data were analyzed in MATLAB (MathWorks Inc., United States) using the following processing steps. First, <*t*> was calculated for every DTOF in a time series after truncating each DTOF at 10% of the ascending side and 1% of the descending side to reduce noise (Liebert et al., 2004).<*t*> was chosen since previous work has shown that it provided a good compromise between activation sensitivity and signal-to-noise ratio (Abdalmalak et al., 2017a). The change in mean time-of-flight (Δ<*t*>) relative to the initial values was calculated, and these time series were corrected for motion artifacts using an algorithm based on a moving standard deviation and spline interpolation (Scholkmann et al., 2010; Metz et al., 2015). The time-courses were detrended to remove slow drifts by filtering with a high-pass filter with a cut-off period of 128 s and smoothed using a hemodynamic response function (full width half maximum = 4 s) to remove fast frequency components, such as those due to arterial pulsation. Next, the two Δ<*t*> time-courses for λ = 760 and 830 nm were converted into changes in concentration of oxy- and deoxy-hemoglobin using sensitivity factors obtained from Monte Carlo simulations. These simulations were generated based on a 10-layer model in which each layer was 0.2 cm thick. At each wavelength, the sensitivity factor for the brain was calculated as the sum of the sensitivity factors for all layers below 1 cm (i.e., layers 5–10) (Kacprzak et al., 2007; Abdalmalak et al., 2017b).

To calculate the changes in the concentrations of oxyhemoglobin (Δ*C*_*HbO*_2__) and deoxyhemoglobin (Δ*C*_*Hb*_), Δ<*t*> was first converted to the corresponding change in the absorption coefficient, Δμ_*a*_(λ), for the two wavelengths (λ = 760 and 830 nm):

(1) $$\Delta \,\mu_{a}\,\left(\lambda \right)=\frac{\Delta \,\left\langle t\right\rangle }{M\,T\,S\,F}=\frac{\left\langle t\right\rangle -{\left\langle t\right\rangle }_{0}}{M\,T\,S\,F}$$

where, MTSF is the sensitivity factor derived from Monte Carlo simulations for Δ<*t*> in the brain. Next, Δμ_*a*_(λ) values determined at 760 and 830 nm were converted to Δ*C*_*HbO*_2__ and Δ*C*_*Hb*_ by:

(2) $$\Delta \,\mu_{a}\,\left(\lambda \right)=\varepsilon_{H\,b\,O_{2}}\,\left(\lambda \right)\,\Delta \,C_{H\,b\,O_{2}}+\varepsilon_{H\,b}\,\left(\lambda \right)\,\Delta \,C_{H\,b}$$

where, ε_*HbO*_2__(λ) and ε_*Hb*_(λ) are the molar extinction coefficients for oxy- and deoxy-hemoglobin, respectively. After preprocessing, signals were averaged across all five trials and across all channels for each question; i.e., the response for each question was reduced to a single average time-course (60 s consisting of two 15 s rest periods and 30 s response period) for oxy- and deoxy-hemoglobin, respectively. Averaging was conducted to improve the signal-to-noise ratio and reduce the chance of detecting false positives based on the assumption that all four channels were interrogating motor-planning areas.

Features (listed in Table 1) were then extracted from the average time-courses for oxyhemoglobin only, since previous work has shown that oxyhemoglobin yields better performance for assessing task-induced brain activation (Mihara et al., 2012; Naseer and Hong, 2013). In order to investigate which combination of features produced the highest accuracy, an LDA and an SVM classifier were used to classify the result using the leave-one-out cross-validation method with all possible unique feature combinations (15 combinations in total). The classifier with the combination of feature(s) that yielded the highest accuracy was used to obtain all the results presented in this study. The code used for the analysis was developed in MATLAB (MathWorks Inc., United States) using functions implemented in the Statistical and Machine Learning Toolbox. Furthermore, a one-way ANOVA was used to determine if there was a significant difference in accuracy between questions (i.e., questions 1–4). Finally, to investigate the effect of the number of cycles on the overall accuracy, the analysis was initially conducted with only the first cycle and then repeated with increasing number of cycles until all five cycles were included.

**TABLE 1:** Features extracted from the oxyhemoglobin time-courses and how each feature was calculated.

Feature	Calculation
Median change in signal (SM)	Difference between the median change during the task (excluding the first 10 s) and the preceding rest period
Signal slope (SS)	Slope of the first 16 s during the task period
Contrast-to-noise ratio (CNR)	Difference between the mean change during the task and the preceding rest period divided by the standard deviation of the rest period
Correlation coefficient (*r*)	Correlation coefficient between the change in the hemoglobin concentration time-courses and the theoretical activation model (i.e., box function convolved with a hemodynamic response function)

## Results

**FIGURE 2:**
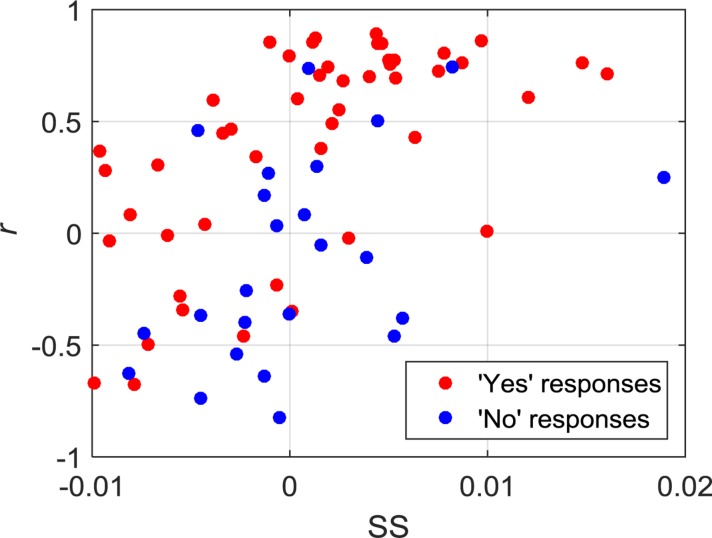
2D feature space showing the relationship between SS and *r* for all of the “yes” and “no” responses.

Of the 21 participants, three had to be excluded due to significant motion artifacts and overall low signal quality. The overall classification accuracy across all included subjects using LDA was 75% with a sensitivity of 83% and specificity of 58%. Similarly, the classification accuracy using SVM was 76% with a sensitivity of 79% and specificity of 71%. Individual classification accuracies using both classifiers are shown in Table 2. The combination of features that produced the highest accuracy using LDA was SM, CNR, and *r*, while SS and *r* contributed the most to the SVM model. Since SVM produced a higher accuracy, it was used for all further analyses. Figure 2 shows the SS and *r* plotted in a 2D feature space for the “yes” and “no” responses in order to visualize the difference between the two responses.

**TABLE 2:** Individual classification results for each participant.

Participant number	LDA Accuracy (%)	SVM Accuracy (%)
1	75	75
2	50	50
3	75	75
4	50	75
5	100	100
6	100	100
7	75	75
8	100	100
9	75	75
10	100	100
11	75	100
12	75	75
13	75	75
14	50	75
15	50	50
16	100	75
17	75	50
18	50	50

**FIGURE 3:**
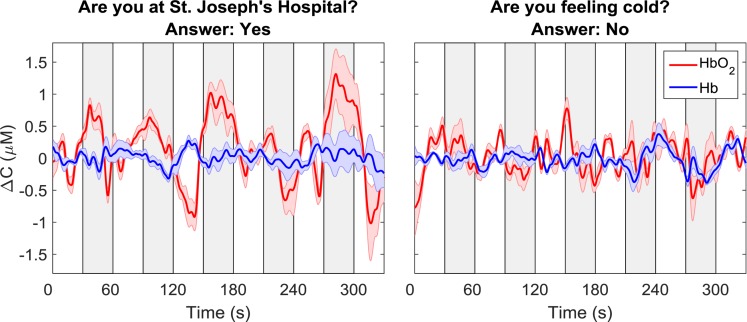
Sample time courses of Δ*C_HbO__2_* and Δ*C_Hb_* for one participant and two questions. Each time course was averaged across data from all four channels. The time course on the left was classified as “yes” while the one on the right was classified as “no.” The gray boxes indicate the response periods. The error bars represent the standard error of mean across channels.

The oxy- and deoxy-hemoglobin time-courses for one participant and for two different questions are shown in Figure 3. For the time-course shown on the left, which corresponded to the question: “Are you at St. Joseph’s Hospital?,” a clear increase in oxyhemoglobin and a concurrent, but smaller, decrease in deoxyhemoglobin can be observed during the response periods. For the second question in which the participant’s response was “no,” there were no noticeable changes in either Δ*C*_*HbO*_2__ or Δ*C*_*Hb*_. As expected, these two questions were classified as “yes” and “no,” respectively.

**FIGURE 4:**
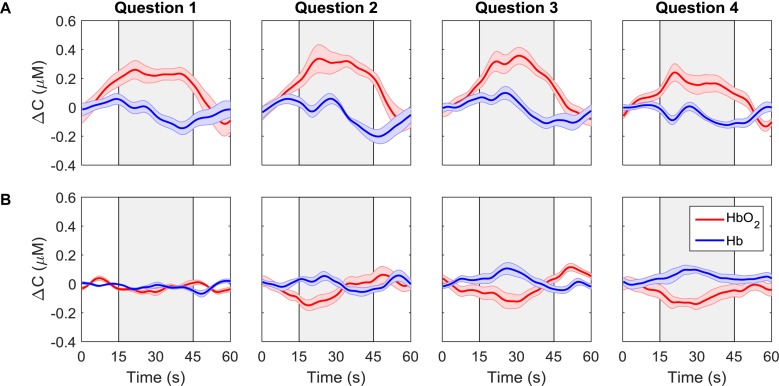
ΔC*_HbO__2_*(red) and ΔC_Hb_ (blue) for each question averaged across all trials, channels, and participants. Each column represents a different question. The first row **(A)** shows the signals that were classified as “yes” while the second row **(B)** shows the signals that were classified as “no.” The gray boxes indicate the response period. The error bars represent the standard error of mean across participants (*n* = 18).

Average time courses of Δ*C*_*HbO*_2__ and Δ*C*_*Hb*_ for each consecutive question are presented in Figure 4. Since the order of the questions was randomized, each subplot does not represent the response to a particular question, but rather the response to all questions asked in one period. For each participant, the time courses were first averaged across trials and channels, resulting in a single time course per question. These time-courses were then averaged across all participants for the “yes” and “no” responses based on the classifier output. The “yes” responses show the expected hemodynamic changes in oxy-and deoxy-hemoglobin, which are absent in the “no” responses.

**FIGURE 5:**
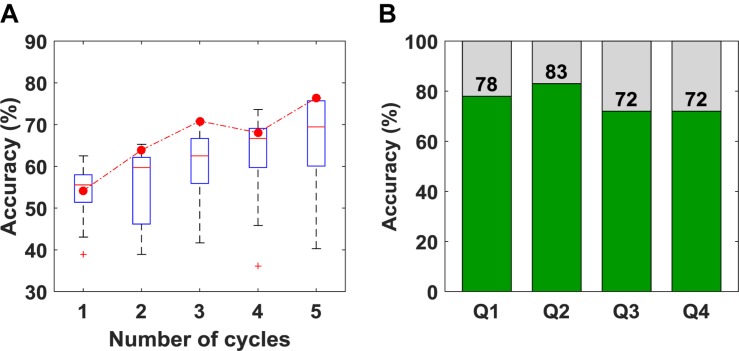
**(A)** Classification accuracy obtained versus the number of cycles used for classification. The box plot shows the variation in accuracy for all 15 unique combinations of features. The red circles represent the accuracy for the set of features that was selected as optimum **(B)** Classification accuracy obtained for questions 1–4 using five cycles for classification.

The overall accuracy of the SVM results is plotted as a function of the number of cycles in Figure 5A. As expected, increasing the number of cycles used for classification improved accuracy. The box-plot in Figure 5A shows variation in accuracy for each cycle for all unique combinations of features (15 in total), and the red circles represent the accuracy obtained using the optimum combination of features for SVM (i.e., SS and *r*). Since the best combination of features was optimized for five cycles, using only one, two or four cycles leads to different sets of optimum features. The classification accuracies for questions one to four are shown in Figure 5B. Once again, the accuracy presented is not for a particular question but based on the order of the questions asked. Although there appear to be differences in accuracy between questions, there were no statistically significant differences.

**FIGURE 6:**
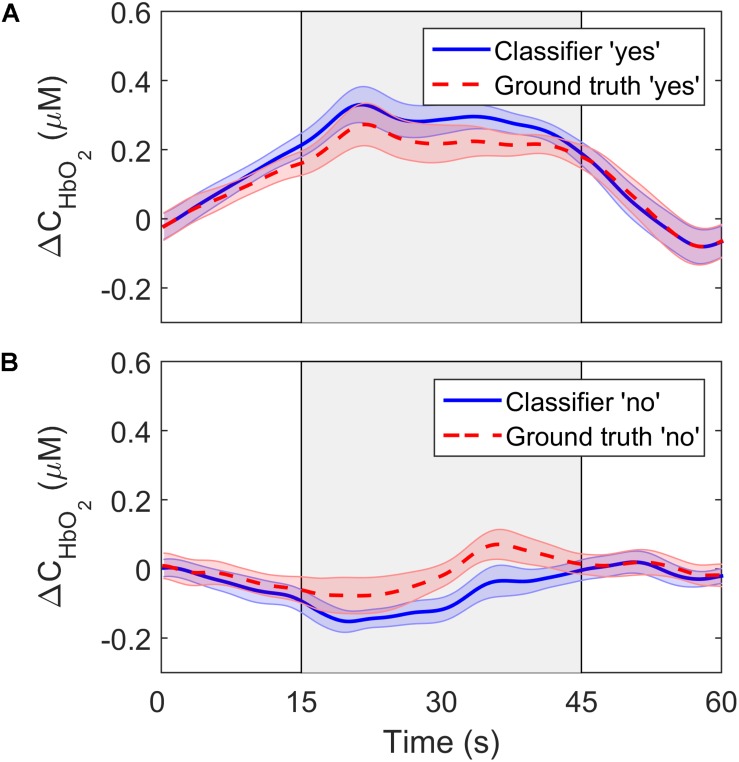
ΔC*_HbO__2_* averaged across channels, trials and participants for **(A)** the “yes” responses and **(B)** the “no” responses. The solid lines show the signals based on the SVM classifier output while the dashed lines represent the ground truth responses. The error bars represent the standard error of mean across participants (*n* = 18).

To further investigate the performance of the SVM classifier, the oxyhemoglobin signals that were classified as “yes” or “no” were averaged together for all trials, channels, participants, and questions. In other words, the oxyhemoglobin time-courses for the “yes” and “no” responses in Figure 4 were averaged together to end up with one time-course for all “yes” responses and one time-course for all “no” responses. In addition, the oxyhemoglobin time-courses for the ground truth responses, i.e., based on the participants’ responses recorded after the study, were averaged together to produce ground-truth “yes” and “no” oxyhemoglobin time-courses. The ground-truth “yes” signal represents the group average for all oxyhemoglobin signals for which participants answered “yes.” Likewise, the ground-truth “no” is the group average for questions where participants answered “no.” These two sets are shown in Figure 6. As expected, the “yes” responses showed an increase in the signal during the response period. Interestingly, the ground truth “no” time-course also showed an unexpected increase in the signal during the response period upon visual inspection. This change was approximately 25% of the maximum change observed for the corresponding “yes” time-course.

The MAP and HR values averaged across the seven participants for MI and rest, respectively, are shown in Table 3. No significant difference between the two conditions was found.

**TABLE 3:** Physiological parameters obtained during motor imagery and rest.

	Rest	Change during MI	Range
MAP (mmHg)	77 ± 8	2 ± 1	−3, 5
HR (bpm)	70 ± 10	3 ± 2	−5, 5

*MAP, Mean arterial pressure; HR, Heart rate.*

## Discussion

The goal of this study was to assess the feasibility of TR-fNIRS as a BCI for mental communication. The study focused on a MI paradigm (i.e., imagine playing tennis) that has been used previously with fMRI to assess residual brain function in DOC patients and to provide rudimentary mental communication (Monti et al., 2010). Furthermore, the detection sensitivity of TR-fNIRS for this tennis imagery task was found to be comparable to fMRI in a cohort of healthy participants (Abdalmalak et al., 2017a). Based on these promising results, the motivation for this study was to evaluate the combination of TR-NIRS and MI for mental communication involving multiple closed-ended questions. A series of four questions was asked of each healthy participant and classification accuracy was assessed for two commonly used machine-learning algorithms (LDA and SVM) (Hong et al., 2018). Both algorithms produced similar accuracies (76% for SVM and 75% for LDA); however, SVM resulted in a better balance between sensitivity and specificity (79% and 71%, respectively) compared to LDA (83% and 58%, respectively). Overall, these estimates of classification accuracy are in-line with previous reports (Naseer and Hong, 2015a) and meet the minimum threshold of 70% for a BCI to be considered effective for communication (Proulx et al., 2018).

Although the classification accuracy is comparable to results from other fNIRS studies involving various activation tasks for mental communication (Naseer and Hong, 2015b), it was less than the accuracy reported in an fMRI study involving the same tennis imagery task (Monti et al., 2010). One possible explanation is related to the challenges of detecting MI by fNIRS due to the presence of hair and the increased scalp-brain distance over the motor-planning areas relative to the frontal regions (Cui et al., 2011). The latter was likely compounded by the observation from fMRI studies that MI-related activation in the SMA frequently occurs at a greater distance from the cortical surface (Monti et al., 2010; Taube et al., 2015). TR detection will help compensate for activation at greater depths (Milej et al., 2019); however, these challenges reflect the lower classification accuracy generally reported for MI compared to tasks that activate the prefrontal cortex (Shin et al., 2017; Qureshi et al., 2017). It should also be noted that the activation contrast elicited by MI is less than for motor execution tasks (Batula et al., 2017), and activation for mental imagery tasks is not detectable in a small subset of participants, typically on the order of 10–15% (Fernández-Espejo et al., 2014). Unlike our previous study (Abdalmalak et al., 2017a), the current study did not include fMRI to confirm detectable MI activation for all participants. This would explain why the sensitivity in the current study (on the order of 80%) was lower than the sensitivity calculated previously when MI activation detected by TR-fNIRS was compared to fMRI results (Abdalmalak et al., 2017a).

While classification accuracy has been the most commonly used metric in fNIRS-BCI studies (Naseer and Hong, 2015a; Rupawala et al., 2018), sensitivity and specificity were also computed in the current study. These are important metrics in BCI applications for evaluating the confidence that can be placed on a measured response. This is relevant to applications involving DOC patients that are aimed at evaluating residual brain function and providing rudimentary mental communication (Peterson et al., 2015). The sensitivity of the LDA and SVM algorithms were similar (83% and 79%, respectively), but specificity was lower for both: 58% for LDA and 71% for SVM. Considering that specificity reflects the ability of the classifier to accurately detect a “no” response, the poorer results indicate that the inherent fluctuations in NIRS time courses were leading to false positives. This is confirmed by the average time courses shown in Figure 6. The ground truth “no” response showed an unexpected signal increase during the response period at approximately the 40-s mark. Similar artifacts are evident in other fNIRS-BCI studies that relied on a stable signal time-course to reflect a “no” response (Naseer et al., 2014), and reflect the challenges of removing all sources of noise in the pre-processing steps, particularly motion artifacts and low-frequency spontaneous oscillation.

There are a number of potential approaches that could be used to improve specificity. The first would be to use an active task for the “no” response as used in fMRI studies (Monti et al., 2010). For example, the “yes” response could be MI, while the “no” response could be a different task that activates brain areas other than the SMA (Bauernfeind et al., 2011). However, it is important to acknowledge that asking patients to perform two complex tasks, such as MI and mental arithmetic, could be challenging. Alternatively, “yes” and “no” responses could be decoded temporally instead of spatially. Bettina and colleagues demonstrated that healthy controls were able to encode at least four distinct answers on a single trial level by performing MI to the temporal prompt corresponding to the desired answer (Sorger et al., 2009; Nagels-Coune et al., 2017). Finally, participants could undergo some form of training to provide some familiarization with using MI for mental communication. None of the participants in this study received training prior to data collection, and it would be valuable to assess if classification accuracy would be improved on a return visit.

A variety of features have been investigated for fNIRS-BCI applications, including mean changes in concentration of oxy- and/or deoxy-hemoglobin, signal slope, the shape of the signal responses (i.e., skewness and kurtosis), et cetera (Naseer and Hong, 2015a). This study included similar features (SM, SS, CNR, see Table 1) as well as the correlation coefficient (*r*) between the HbO_2_ time course and the model function obtained by convolving a box function representing task periods with the hemodynamic response function. For features such as the SS, there is some ambiguity regarding the appropriate period for calculating the signal slope. In this study, the slope was calculated over 16 s; however, a shorter period could have been selected based on the hemodynamic response function that peaks at 7 s. To investigate the potential impact of reducing the period, the analysis was repeated for a slope calculated over the first 7 s of the task period. This change resulted in a small reduction (4%) in the accuracy for the SVM algorithm, which is likely due to variability in the peak hemodynamic response between individuals (see Figure 4 channel 2 for example).

A limitation with using features like *r* is the large amount of data required to obtain a reliable estimate. This was confirmed by the results presented in Figure 5A, showing the expected improvement in accuracy as the number of task cycles increased from one to five. The obvious disadvantage of using all five cycles is that the approach is not suitable for real-time applications. However, for our goal of applying this methodology to helping evaluate consciousness in DOC patients, this is not a concern. Furthermore, *r* was the only feature common to both the final SVM and LDA algorithms, highlighting its value for optimizing classification accuracy.

One of the challenges with generic BCIs is inter-subject variability. Individual accuracies in this study varied from chance level to classifying all four questions correctly (Table 2). Psycho-physiological factors, such as attention and memory load, could contribute to the observed inter-subject variability. It has also been suggested that females, individuals over the age of 25, and those who play instruments are likely to perform better at mental imagery tasks (Randolph, 2012; Ahn and Jun, 2015). In this study, there was an imbalance between males and females; however, the total number of participants was not sufficient to assess if sex or age could have affected task performance. Additionally, it is known that task-induced changes in HR and MAP can potentially degrade the fNIRS signals, leading to false positives (Caldwell et al., 2016). To assess this potential source of error, HR and MAP were measured in seven participants performing MI in the same block design used in the BCI experiments, and no significant difference between the two conditions was found.

Another common challenge with most BCIs for mental communication is the trade-off between accuracy and the time delay before defining a response. In general, the greater the number of trials acquired prior to feature extraction and classifying the signals, the greater the SNR and hence the overall classification accuracy. BOLD-dependent modalities such as fMRI and fNIRS are inherently slow as the hemodynamic response peaks around 7 s post-stimulus. In contrast, EEG, which directly measures neuronal activity, can provide much faster responses. However, the majority of EEG-based BCIs do not display the results in real-time since most of these are classifier-based and often take time to judge and classify the signals to ensure accuracy. It is important to emphasize that the intended goal of our TR-fNIRS BCI is to assess residual awareness in DOC patients and therefore our protocol is intentionally long in order to maximize the confidence in the recorded responses.

In conclusion, this work highlights the potential of TR-fNIRS as a BCI for mental communication. Our approach focused on using a few detection channels that targeted specific brain regions known to be involved with MI. This is a relatively simple approach that is well suited for BCI applications without the need for training (Abdalmalak et al., 2017b). Our results indicate that the current method provides sufficient classification accuracy for clinical application. Since the technology is readily adaptable to other tasks/brain regions, incorporating separate active tasks for a “no” response could be considered to further improve the accuracy. In addition, the use of more sophisticated classifiers could be explored to further enhance performance.

## Data Availability Statement

The datasets generated for this study are available upon request to the corresponding author.

## Ethics Statement

The studies involving human participants were reviewed and approved by the Research Ethics Board at Western University, which complies with the guidelines of the Tri-Council Policy Statement (TCPS): Ethical Conduct for Research Involving Humans. The patients/participants provided their written informed consent to participate in this study.

## Author Contributions

AA, AO, and KS contributed to the conception and design of the study. AA, LY, and DM developed the TR-fNIRS system used in this study. AA, DM, and KS contributed to the methodology and data acquisition. AA and DM with inputs from AK, MD, and KS carried out the fNIRS data analysis. AA wrote the manuscript with inputs from all the authors. DM, LY, AK, MD, AO, and KS contributed to critically revising the manuscript for important intellectual content. AO and KS secured funding for the study. All authors approved the final version of the manuscript for publication.

## Conflict of Interest

The authors declare that the research was conducted in the absence of any commercial or financial relationships that could be construed as a potential conflict of interest.
